# Comparative Analysis of Primary Sarcopenia and End‐Stage Renal Disease–Related Muscle Wasting Using Multi‐Omics Approaches

**DOI:** 10.1002/jcsm.13749

**Published:** 2025-04-10

**Authors:** Daiki Setoyama, Dohyun Han, Jingwen Tian, Ho Yeop Lee, Hyun Suk Shin, Ha Thi Nga, Thi Linh Nguyen, Ji Sun Moon, Hyo Ju Jang, Evonne Kim, Seong‐Kyu Choe, Sang Hyeon Ju, Dae Eun Choi, Obin Kwon, Hyon‐Seung Yi

**Affiliations:** ^1^ Department of Clinical Chemistry and Laboratory Medicine Kyushu University Hospital Fukuoka Japan; ^2^ Proteomics Core Facility Seoul National University Hospital Biomedical Research Institute Seoul South Korea; ^3^ Laboratory of Endocrinology and Immune System Chungnam National University School of Medicine Daejeon South Korea; ^4^ Department of Medical Science Chungnam National University School of Medicine Daejeon South Korea; ^5^ Department of Biomedical Sciences, BK21 FOUR Biomedical Science Program Seoul National University College of Medicine Seoul South Korea; ^6^ Department of Medicine, Graduate School Wonkwang University Iksan South Korea; ^7^ Sarcopenia Total Solution Center Wonkwang University Iksan South Korea; ^8^ Department of Internal Medicine Chungnam National University School of Medicine Daejeon South Korea; ^9^ Department of Biochemistry and Molecular Biology Seoul National University College of Medicine Seoul South Korea; ^10^ Genomic Medicine Institute, Medical Research Center Seoul National University Seoul South Korea

**Keywords:** end‐stage renal disease, metabolomics, multi‐omics, proteomics, sarcopenia

## Abstract

**Background:**

Age‐related primary sarcopenia and end‐stage renal disease (ESRD)–related muscle wasting are discrete entities; however, both manifest as a decline in skeletal muscle mass and strength. The etiological pathways differ, with aging factors implicated in sarcopenia and a combination of uremic factors, including haemodialysis, contributing to ESRD‐related muscle wasting. Understanding these molecular nuances is imperative for targeted interventions, and the integration of proteomic and metabolomic data elucidate these intricate processes.

**Methods:**

We generated detailed clinical data and multi‐omics data (plasma proteomics and metabolomics) for 78 participants to characterise sarcopenia (*n* = 28; mean age, 72.6 ± 7.0 years) or ESRD (*n* = 22; 61.6 ± 5.5 years) compared with controls (*n* = 28; 69.3 ± 5.7 years). Muscle mass was measured using bioelectrical impedance analysis and handgrip strength. Five‐times sit‐to‐stand test performance was measured for all participants. Sarcopenia was diagnosed in accordance with the 2019 Consensus Guidelines from the Asian Working Group for Sarcopenia. An abundance of 234 metabolites and 722 protein groups was quantified in all plasma samples using liquid chromatography with tandem mass spectrometry.

**Results:**

Muscle mass, handgrip strength and lower limb muscle function significantly lower in the sarcopenia group and the ESRD group compared with those in the control group. Metabolomics revealed altered metabolites, highlighting exclusive differences in ESRD‐related muscle wasting. Metabolite set enrichment analysis revealed the involvement of numerous metabolic intermediates associated with urea cycle, amino acid metabolism and nucleic acid metabolism. Catecholamines, including epinephrine, dopamine and serotonin, are significantly elevated in the plasma of patients within the ESRD group. Proteomics data exhibited a clearer distinction among the three groups compared with the metabolomics data, particularly in distinguishing the control group from the sarcopenia group. The ciliary neurotrophic factor receptor was top‐ranked in terms of the variable importance of projection scores. Plasma AHNAK protein levels was higher in the sarcopenia group but was lower in the ESRD group. Proteomic set enrichment analysis revealed enrichment of several pathways related to sarcopenia, such as hemopexin, defence response and cell differentiation, in sarcopenia group. Multi‐omic integration analysis revealed associations between relevant metabolites, including catecholamines, and a group of annotated proteins in extracellular exosomes.

**Conclusions:**

We identified distinct multi‐omic signatures in individuals with ESRD or sarcopenia, providing new insights into the mechanisms underlying ESRD‐related muscle wasting, which differ from primary sarcopenia. These findings may support interventions for context‐dependent muscle loss and contribute to the development of targeted treatments and preventive strategies for muscle wasting.

## 1 Introduction

Age‐related sarcopenia and end‐stage renal disease (ESRD)–related muscle wasting represent two distinct forms of skeletal muscle mass loss, coupled with decreased muscle strength and physical performance. Primary sarcopenia is characterized by the progressive loss of skeletal muscle mass and strength associated with aging [1], whereas muscle wasting in patients with ESRD can be attributed to a combination of uremic factors and haemodialysis [2]. These two types of muscle loss disorders are induced by very different factors but ultimately result in similar muscle atrophy and dysfunction owing to an imbalance between anabolic and catabolic factors, which may be aggravated further by malnutrition, physical inactivity, metabolic perturbation, chronic inflammation, oxidative stress and hormonal imbalances [1, 2].

Understanding the molecular and cellular differences between primary sarcopenia and ESRD‐related muscle wasting is crucial to develop targeted interventions and improve patient care. Although the genetic architecture and molecular mechanisms underlying sarcopenia remain largely unclear, recent advancements in multi‐omic techniques may have provided powerful tools to comprehensively analyse the molecular changes associated with the pathophysiology of sarcopenia [3]. Unlike genomic DNA and transcripts, proteins and metabolites offer a more relevant perspective for biomarker research, as they can reach the endpoint of the ‘omics cascade’ and provide a different insight into the disease phenotype. However, not all technologies can capture the entire view of the biological complexity of this disorder, whereas multi‐omic analyses may reveal new insights [3]. Therefore, the integration of proteomics and metabolomics data from individuals diagnosed with primary sarcopenia and ESRD can comprehensively elucidate these two distinct conditions and identify potential therapeutic targets.

In this study, we aimed to compare healthy controls with participants with sarcopenia and ESRD‐related muscle wasting using a multi‐omic approach. Using state‐of‐the‐art techniques, we analysed the proteomic and metabolomic data derived from plasma samples to characterize distinct molecular patterns, signalling pathways and biological processes. The application of these techniques in this study represents a significant advancement in elucidating the molecular complexity of primary sarcopenia and ESRD‐related muscle wasting. Through an unbiased comprehensive multi‐omic analysis, we provide insights into age‐ or ESRD‐related metabolic alterations and identify potential targets for treatments specific to each condition.

## 2 Methods

### 2.1 Study Participants

This cross‐sectional study was conducted on a cohort of ambulatory community‐dwelling older adults. The study cohort comprised Korean individuals who received follow‐up care at the Department of Internal Medicine of Chungnam National University Hospital (CNUH, Daejeon, South Korea) between January 2021 and January 2023. These participants visited the clinic for the management of non‐functional thyroid diseases, hypertension, dyslipidaemia, type 2 diabetes, osteopenia, osteoporosis or haemodialysis and were not residents of nursing homes or inpatient facilities. The exclusion criteria included a history of ongoing cancer or a cancer‐free period of < 5 years, psychiatric disease, malabsorption disorder (including diabetic gastropathy or a history of bowel resection), symptomatic heart failure, pregnancy, acute bacterial or viral infection, chronic obstructive pulmonary disease, neurodegenerative disease or the requirement of a wheelchair for mobility **(**Figure S1 **)**. After excluding ineligible participants, non‐fasting blood samples were collected from 78 eligible individuals. All patients with ESRD underwent conventional haemodialysis three times per week for approximately 4 h. Blood samples were collected from participants before the initiation of haemodialysis without extra puncture. This study was approved by the Institutional Review Board of Chungnam National University Hospital (CNUH‐2020‐10‐019‐005), and written informed consent was obtained from all the participants.

### 2.2 Sarcopenia Assessment

Demographic characteristics and medical/surgical histories were obtained through comprehensive interviews and review of medical records by experienced nurses. Body composition, which included muscle mass (whole‐body lean body mass [LBM] minus bone mineral content), was assessed using a bioelectrical impedance analyser (InBody 270 Body Composition Analyser; InBody USA, Cerritos, CA, USA) at 1, 5, 50, 250, 500 and 1000 kHz.

Appendicular skeletal muscle mass (ASM) was defined as the combined muscle mass of all four limbs, and the skeletal muscle index (SMI) was calculated as ASM divided by height squared (ASM/m^2^). Handgrip strength (HGS) of the dominant side was measured using a Smedley‐type dynamometer (Takei T.K.K.5401 GRIP‐D handgrip dynamometer; Takei Scientific Instruments Co., Ltd., Tokyo, Japan). For patients undergoing haemodialysis, muscle strength was assessed based on HGS on the side without the haemodialysis access (arteriovenous fistula or graft). The participants were instructed to sit comfortably, bend their elbows at 90° and squeeze the dynamometer with maximal effort. This test was conducted three times at 1‐min intervals, and the highest recorded value was used for analysis.

Physical performance was assessed using the Short Physical Performance Battery (SPPB), which comprises standardized performance tests including gait speed in a 4‐m walk, a five‐times sit‐to‐stand test (5TSTS) to evaluate coordination and strength, and a tandem test to assess static balance. The tandem test was performed in three different positions: side by side, semi‐tandem and full tandem. The participants were instructed to maintain each position for > 10 s, and the recorded time (in seconds) at which they successfully held each position was noted.

Sarcopenia was diagnosed based on the 2019 Consensus Guidelines from the Asian Working Group for Sarcopenia [4]. Participants were classified as having sarcopenia if they exhibited low ASM measured by bioelectrical impedance analysis (< 7.0 kg/m^2^ for men and < 5.7 kg/m^2^ for women) and low muscle strength (HGS < 28 kg for men and <18 kg for women).

### 2.3 Measurements of Plasma Metabolome and Proteome With Mass Spectrometer

Additional methods are provided as supplementary materials.

### 2.4 Data Processing and Statistical Analysis of Omic Data

The metabolome and proteome data were analysed using a partial least squares discriminant analysis (PLS‐DA) in MetaboAnalyst 6.0 for feature extraction and discriminant analysis [5]. The initial step in the data processing was normalization by autoscaling, which entailed mean‐centring and scaling each variable to a unit variance to minimize the influence of differences in metabolite signal ranges. For the proteome data, preprocessing was performed using the Perseus software [6]. Initially, log2 transformation was applied due to the skewed data distribution. Valid values were filtered using proteins with a minimum of 70% quantified values in at least one group. Missing values were imputed based on a normal distribution (width = 0.3, downshift = 1.8) to simulate the signals of low‐abundance proteins. The preprocessed matrix was then subject to statistical analysis.

A PLS‐DA was performed with the optimal number of components selected based on 10‐fold cross‐validation within the platform. The performance of the model was evaluated using *R* ^2^ and *Q* ^2^ metrics to assess the explained variance and the predictive ability of the model, respectively. The variable importance in projection (VIP) scores were calculated to identify the most discriminative metabolites. Metabolites and proteins with VIP scores greater than 1.0 were considered significant contributors to the differentiation between the groups. To ensure the robustness of the findings, the model was validated using permutation tests. The stability of the PLS‐DA model was further assessed through repeated cross‐validation. A hierarchical cluster analysis was performed by the MetaboAnalyst based on metabolites or proteins with VIP value > 1 for heat map visualization.

In proteome data, the quantitative protein intensities spanned eight orders of magnitude across all samples, with the top 10 most abundant proteins contributing 48.8% of all plasma protein abundances in our datasets. The reproducibility of the proteomic analysis was assessed by analysing the abundance of correlated proteins across the entire measurement range. Correlation results showed that the average Pearson's correlation values of the quantified protein signals between individual replicates were 0.862, 0.831 and 0.852 in the control, sarcopenia and ESRD groups, respectively. The average correlation coefficient between the sarcopenia and control groups was 0.815.

### 2.5 Multi‐Omic Data Integration

To investigate the integrative relationships between metabolome and proteome datasets, we employed the Data Integration Analysis for Biomarker discovery using Latent cOmponents (DIABLO) approach [7], implemented in the mixOmics package (version 6.25.1) in R (version 4.4.0) [8]. DIABLO is a multivariate method designed for the analysis of multi‐omic datasets, enabling simultaneous integration and exploration of multiple data types. Prior to analysis, both metabolome and proteome datasets were auto‐scaled. DIABLO was performed using the block.splsda function in mixOmics. The metabolome and proteome datasets were treated as distinct blocks, with the primary objective of identifying the most correlated variables across these omic layers that discriminate between control, ESRD and sarcopenia groups. The tuning of DIABLO parameters, including the number of components and the number of variables to select from each block, was achieved through cross‐validation. We used 10‐fold cross‐validation, repeated 10 times, to determine the optimal number of components and the number of variables per component that minimized classification error. The results were visualized using various plotting functions in mixOmics, such as ‘plotDiablo’ in Figure 8B, which provides a graphical representation of the correlations between the omic datasets, and ‘circosPlot’ in Figure 8D, which illustrates the associations between selected features across blocks. Additionally, the network function was utilized to visualize the relationships between the variables within and across omic datasets.

### 2.6 STRING Network and Gene Ontology (GO) Analysis

Following DIABLO analysis, proteins identified as potential biomarkers were further analysed for their functional relevance. We employed the STRING database (version 12.0) to perform protein–protein interaction network analysis [9]. The identified proteins were mapped onto the STRING network, and interaction scores were calculated to assess the strength of their associations. Highly interconnected proteins within the network, or those with strong interaction scores, were considered for further functional annotation. Subsequently, GO enrichment analysis was conducted on the proteins of interest to categorize them into biological processes, molecular functions and cellular components. Chemical–protein interactions in the largest network were assessed by MBROLE3 [10].

### 2.7 Sample Size Calculation

In this pilot trial, we determined the sample size based on previously published guidelines for pilot trial sample size calculations, which recommended a minimum of 20 patients in total [11]. In addition, we performed power analysis using the G*power software [12]. The power reaches an acceptable level (0.8) at a sample size of approximately 78. Taking into account potential dropouts, the final sample size was set at 28 for the control group, 28 for patients with sarcopenia and 22 for patients with ESRD, which meets the minimum sample size standard.

### 2.8 Statistical Analysis

Continuous datasets are reported as the mean ± SD, except if explicitly stated otherwise. One‐way analysis of variance with Tukey's multiple comparison test was used to analyse demographic data using the Prism software version 8.3.0 (GraphPad, San Diego, CA, USA), after confirming the normal distribution of each dataset. The ‘ggstatsplot’ creates graphics with details from statistical tests included in the plots themselves. The LabSolutions LC–MS software (Shimadzu, Japan) and the R software version 4.1.0 (R Project for Statistical Computing, Vienna, Austria) were employed for metabolomic data processing. The false discovery rate (FDR) with the Benjamini–Hochberg procedure was used to control Type I error in multiple tests for analysis of metabolomic and proteomic data. *p* < 0.05 was considered to indicate statistical significance.

## 3 Results

### 3.1 Clinical and Functional Characteristics of the Study Participants

**FIGURE 1:**
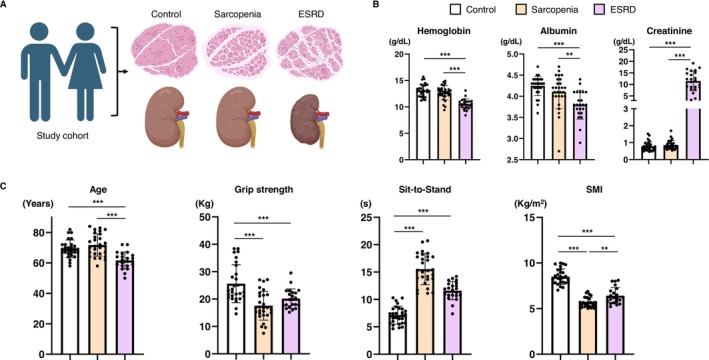
Serum chemical parameters and muscle mass and strength of study participants. (A) Introduction of study population including the control, sarcopenia and ESRD groups. The figure was created with BioRender.com. (B) Serum concentration of haemoglobin, albumin and creatinine in the participants. (C) Age, handgrip strength, five‐times sit‐to‐stand test and SMI of the participants. Data are expressed as means ± standard deviation. *p < 0.05, **p < 0.01. (B, C: ordinary one‐way analysis of variance with Tukey's multiple comparison test). ESRD, end‐stage renal disease; SMI, skeletal muscle index.

We generated plasma proteomic and metabolomic data from totally 78 participants (Figure 1A). We carefully phenotyped the participants by measuring their clinical, physical and serum chemical characteristics (Figure 1). This study enrolled 28 control participants aged 62–82 years (mean age: 69.3 ± 5.7 years; eight men and 20 women), 28 participants with primary sarcopenia aged 62–84 years (72.6 ± 7.0 years; eight men and 20 women) and 22 patients with ESRD aged 50–72 years (61.6 ± 5.5 years; seven men and 15 women). Those with ESRD had significantly lower serum haemoglobin and albumin levels than controls or participants with sarcopenia but exhibited remarkably higher serum creatinine levels (Figure 1B). HGS, 5TSTS performance and SMI were measured for all the participants. Participants with sarcopenia or ESRD showed significantly lower muscle strength and mass than the controls (Figure 1C).

### 3.2 Metabolomic Data: Identification of Candidate Metabolites as Biomarkers for Primary Sarcopenia and ESRD‐Related Muscle Wasting

**FIGURE 2:**
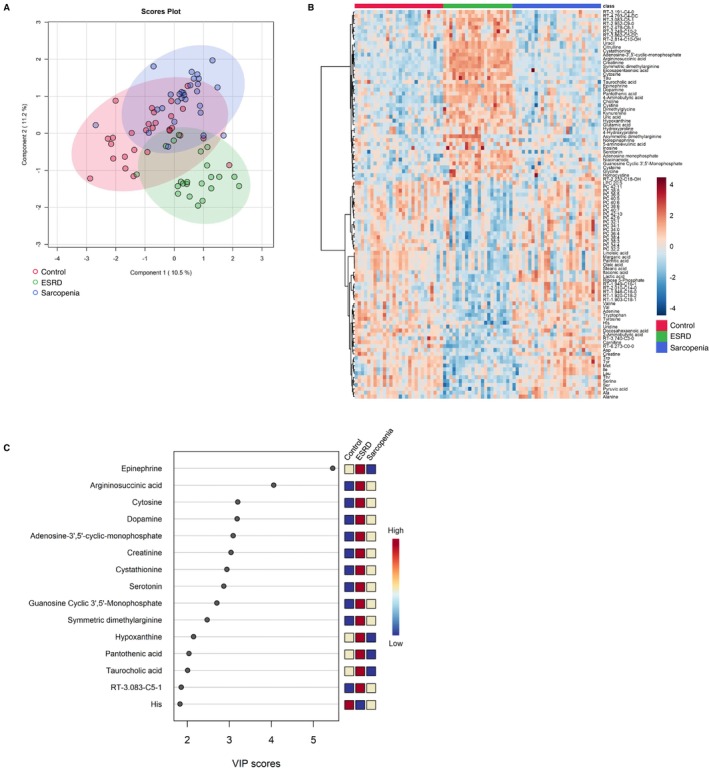
Comparison of plasma metabolite profiles among controls, participants with sarcopenia and patients with ESRD. (A) Partial least square discriminant analysis of plasma metabolite data from all participants. The numbers in parentheses show the contribution rates. The red, green and blue dots indicate controls (*n* = 28), patients with ESRD (*n* = 22) and participants with sarcopenia (*n* = 28), respectively. (B) Visualization of significantly differentiated plasma metabolomic data among control, ESRD and sarcopenia groups in a heatmap. (C) VIP score plot of the metabolites that differed among control, ESRD group and sarcopenia groups. ESRD, end‐stage renal disease; VIP, variable importance in projection.

**FIGURE 3:**
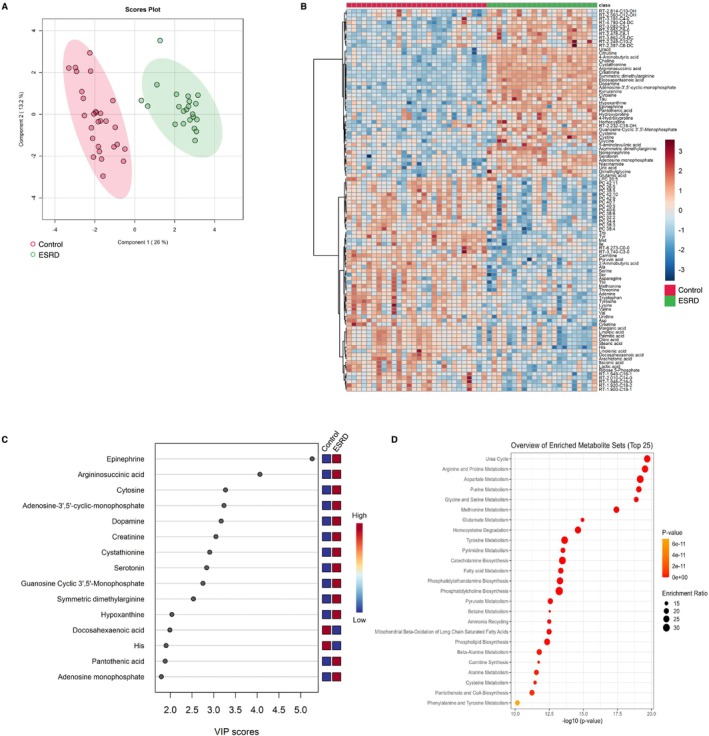
Comparison of plasma metabolite profiles between controls and patients with ESRD. (A) Partial least square discriminant analysis of plasma metabolite data from all participants. The numbers in parentheses show the contribution rates. The red and green dots indicate controls (*n* = 28) and patients with ESRD (*n* = 22), respectively. (B) Visualization of significantly differentiated plasma metabolomic data between control and ESRD groups in a heatmap. (C) VIP score plot of the metabolites that differed between control and ESRD groups. (D) Metabolite set enrichment analysis based on the altered metabolites between the control and ESRD groups. ESRD, end‐stage renal disease; VIP, variable importance in projection.

**FIGURE 4:**
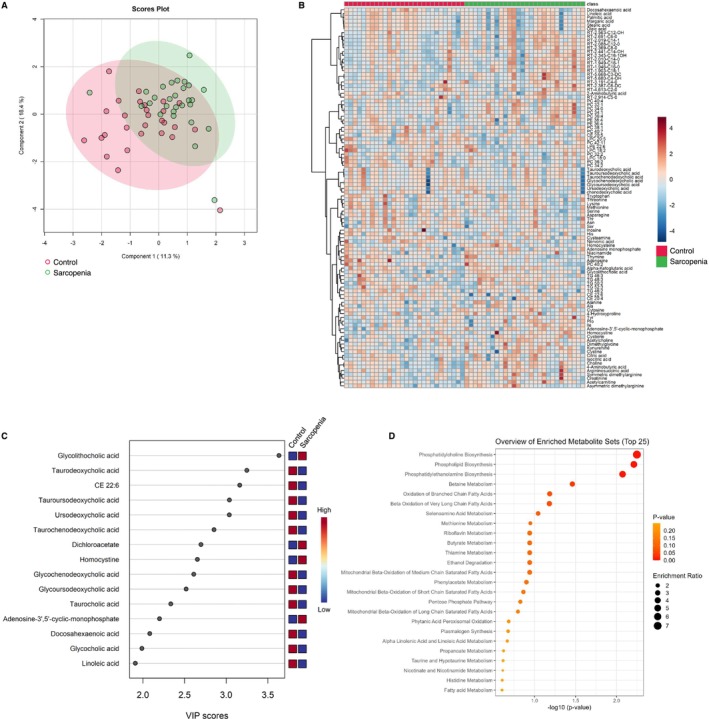
Comparison of plasma metabolite profiles between controls and participants with sarcopenia. (A) Partial least square discriminant analysis of plasma metabolite data from all participants. The numbers in parentheses show the contribution rates. The red and green dots indicate controls (*n* = 28) and participants with sarcopenia (*n* = 28), respectively. (B) Visualization of significantly differentiated plasma metabolomic data between control and sarcopenia groups in a heatmap. (C) VIP score plot of the metabolites that differed between the control and sarcopenia groups. (D) Metabolite set enrichment analysis based on the altered metabolites between the control and sarcopenia groups. VIP, variable importance in projection.

We generated metabolomic data to evaluate the systemically altered metabolites in the peripheral blood of the 78 study participants and quantified the abundance of 234 metabolites (Table S1). Using multivariate information, a PLS‐DA was performed to differentiate the three groups and identify the plasma metabolites that characterized each group. The score plot revealed an ambiguous separation, with a vague and difficult‐to‐distinguish border between the controls and sarcopenia group. Meanwhile, the ESRD group exhibited a clearer separation than the other two groups (Figure 2A). The metabolite heatmap depicts the characteristic expression levels within each group (Figure 2B), and the variable importance in projection (VIP) ranking highlights the metabolites contributing to the discriminant analysis (Figure 2C). In the two‐group discriminant analysis between controls and ESRD group (Figure 3A), differentially expressed metabolites were identified (Figure 3B), clarifying the key metabolites associated with the underlying molecular mechanisms related to renal dysfunction and haemodialysis (creatinine, symmetric dimethylarginine and docosahexanoic acid; Figure 3C). A metabolite set enrichment analysis (MSEA) revealed the involvement of numerous metabolic intermediates associated with urea cycle, amino acid metabolism and nucleic acid metabolism (Figure 3D). Interestingly, catecholamines, including epinephrine, dopamine and serotonin, were significantly elevated in the sera of patients within the ESRD group (Figure 3B,C). The two‐group discriminant analysis of controls and the sarcopenia group (Figure 4A,B) revealed that bile acids were top‐ranked in the VIP scores (Figure 4C) and phospholipid metabolism ranked high in the enrichment analysis (Figure 4D), despite unclear boundaries in the PLS‐DA score plots.

### 3.3 Proteomic Data: Identification of Candidate Proteins as Biomarkers for Primary Sarcopenia and ESRD‐Related Muscle Wasting

**FIGURE 5:**
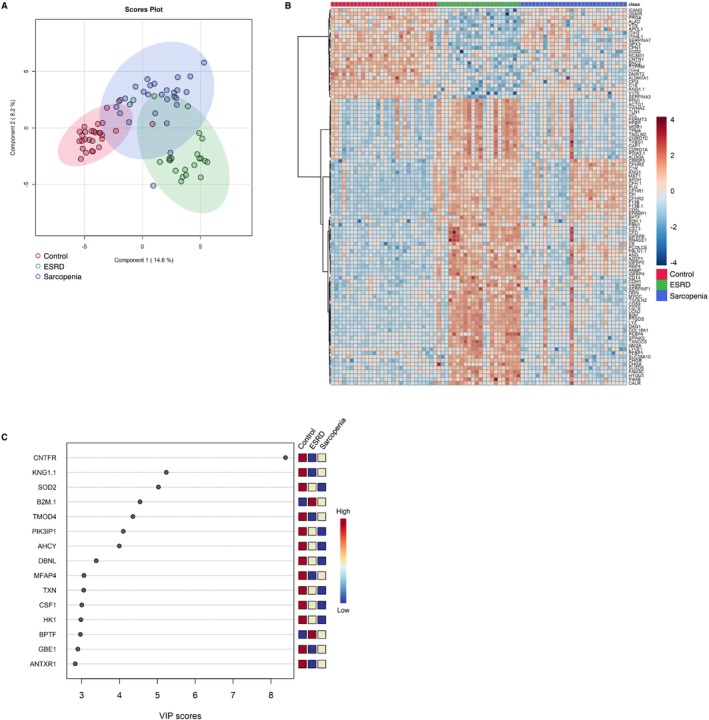
Comparison of plasma proteome profiles among controls, participants with sarcopenia and patients with ESRD. (A) Principal component analysis of plasma protein data from all participants. The numbers in parentheses show the contribution rates. The red, green and blue dots indicate controls (*n* = 28), patients with ESRD (*n* = 22) and participants with sarcopenia (*n* = 28), respectively. (B) Visualization of significantly differentiated plasma proteomic data among the control, ESRD and sarcopenia groups in a heatmap. (C) VIP score plot of the proteins that differed among the control, ESRD and sarcopenia groups. ESRD, end‐stage renal disease; VIP, variable importance in projection.

**FIGURE 6:**
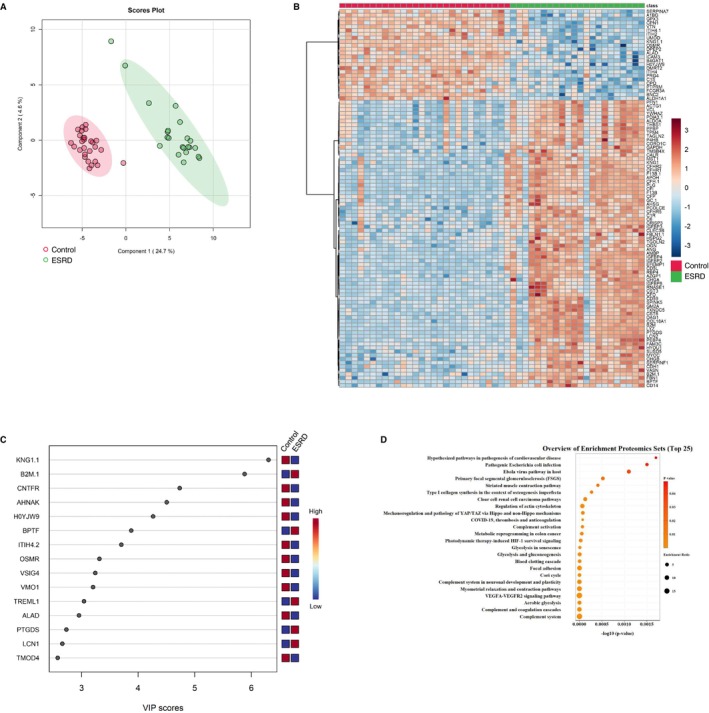
Comparison of plasma proteome profiles between controls and patients with ESRD. (A) Principal component analysis of plasma protein data from all participants. The numbers in parentheses show the contribution rates. The red and green dots indicate controls (*n* = 28) and patients with ESRD (*n* = 22), respectively. (B) Visualization of significantly differentiated plasma proteomic data between the control and ESRD groups in a heatmap. (C) VIP score plot of the proteins that differed between the control and ESRD groups. (D) Proteomic set enrichment analysis based on the altered proteins between the control and ESRD groups. ESRD, end‐stage renal disease; VIP, variable importance in projection.

**FIGURE 7:**
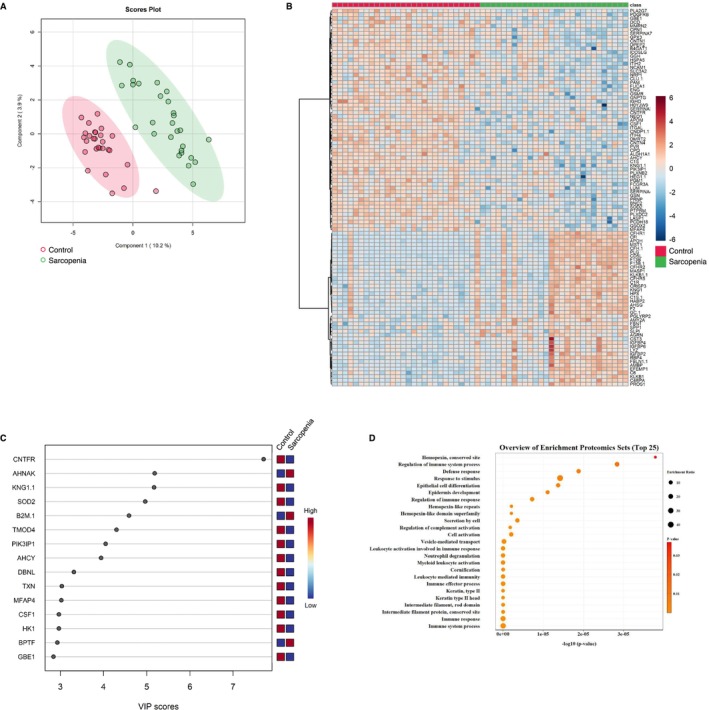
Comparison of plasma proteome profiles between controls and participants with sarcopenia. (A) Principal component analysis of plasma protein data from all participants. The numbers in parentheses show the contribution rates. The red and green dots indicate controls (*n* = 28) and participants with sarcopenia (*n* = 28), respectively. (B) Visualization of significantly differentiated plasma proteomic data between the control and sarcopenia groups in a heatmap. (C) VIP score plot of the proteins that differed between the control and sarcopenia groups. (D) Proteomic set enrichment analysis based on the altered proteins between the control and sarcopenia groups. VIP, variable importance in projection.

To delineate the blood proteome disparities among the three groups, we analysed plasma samples from 78 study participants using quantitative proteomic analysis with DIA. A total of 722 protein groups were quantified in all plasma samples (Table S2). PLS‐DA revealed clear differences among the three groups compared with the metabolomic data (Figure 5A). The protein heatmap depicted the characteristic expression levels within each group (Figure 5B). In the PLS‐DA model, corresponding VIP scores were calculated to assess the identification performance of the investigated proteins. The top 15 proteins with the highest VIP scores in PLS‐DA are presented in Figure 5C. In the two‐group discriminant analysis of controls and the ESRD group (Figure 6A), 102 differentially expressed proteins were identified (Figure 6B). Several of the top 15 proteins of the PLS‐DA VIP list (Figure 6C) were associated with renal dysfunction and haemodialysis (Figure 6D). Compared with the metabolomic data, discriminant analysis of controls and the sarcopenia group revealed a clear separation between the two groups (Figure 7A,B). Interestingly, the ciliary neurotrophic factor receptor (CNTFR), a genetic factor associated with sarcopenia [13], was top‐ranked in the VIP scores (Figures 7C and 5C). Moreover, plasma AHNAK protein was enriched in the sarcopenia group but depleted in the ESRD group. Several pathways related to sarcopenia, such as hemopexin, defence response and cell differentiation, were enriched (Figure 7D).

### 3.4 Integrative Analysis of Metabolomic and Proteomic Data

**FIGURE 8:**
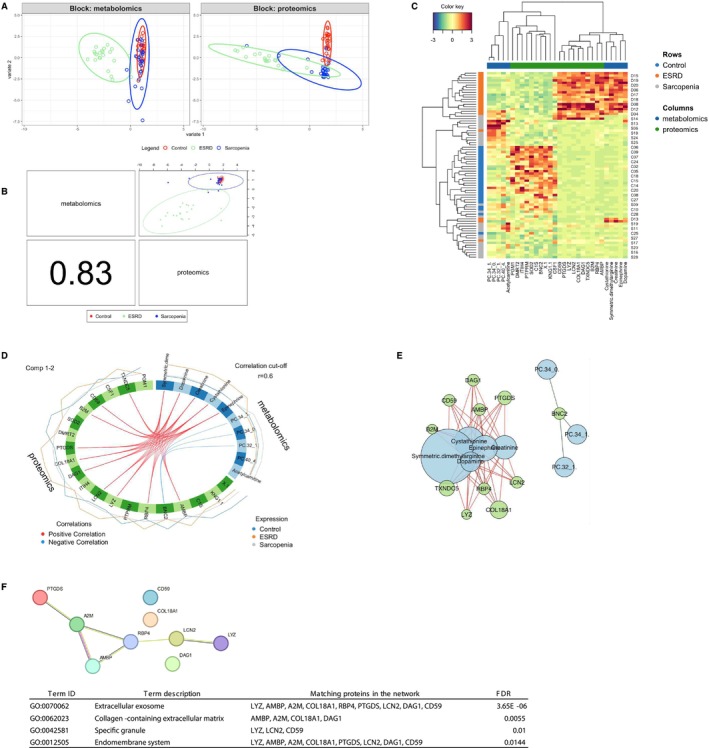
Integrative analysis of metabolome and proteome data with the DIABLO framework. (A) Sample plot of sparse PLS‐DA for each dataset. (B) Sample scatterplot displaying the first component in each data set and the Pearson correlation between each component. (C) Clustered heatmap of the multi‐omics signature. Samples are represented in rows, selected variables on the first component in columns. (D) Circos plot showing the positive (red line) and negative (blue line) correlation (*r* > 0.6) between selected variables on the first two components. (E) Network analysis between selected variables for metabolites (blue) and proteins (green). (F) STRING network and Gene Ontology (GO) analysis of the largest protein cluster observed in (E). ESRD, end‐stage renal disease; FDR, false discovery rate.

To investigate the interrelationships between the metabolome and proteome profiles and identify contributing factors that differentiate between the three distinct groups, we conducted an integrative inter‐omic analysis using the DIABLO. The sample plot with the two components displayed superior discrimination of the three groups based on the proteome data compared with the metabolome data (Figure 8A), confirming previous results (see Figures 2A and 5A). The sample plot with the first component showed a high degree of correlation between the omic data (Figure 8B). The clustered heatmap demonstrated the classification of the three groups based on the top 30 signatures (10 from the metabolome and 20 from the proteome) identified in the first component (Figure 8C). Of these, factors with inter‐omic correlation coefficients greater than 0.6 were illustrated in the circosPlot (Figure 8D) and two clusters were visualized in a relevance network plot (Figure 8E). The largest cluster in the metabolite‐protein network comprised a group of proteins associated with catecholamines, such as dopamine and epinephrine. These were visualized using a STRING network analysis, and a GO analysis revealed that these proteins are annotated as components of extracellular exosomes (Figure 8F). Notably, this metabolite–protein interaction was found to characterize the ESRD group (Figure 8C). In addition, chemical–protein interactions in the largest network were assessed by MBROLE3, which allows the analysis of indirect annotations extracted from the scientific literature and curated chemical–protein associations. Interestingly, this analysis showed that five metabolites were highly associated with oxidoreductase (Figure S2). Of the 10 proteins involved in the network, PTGDS and TXNDC5 were classified as having intramolecular oxidoreductase activity based on the molecular function of GO.

## 4 Discussion

This study explored the altered plasma proteins and metabolites in individuals with ESRD or sarcopenia. Mass spectrometry analysis of the proteome and metabolome revealed concerted sarcopenia‐ or ESRD‐associated perturbations. In our metabolomic analysis, the plasma metabolites involved in sympathetic activation were higher in the ESRD group than in the other groups. Pathway analysis of the metabolome data revealed enriched metabolites related to the urea cycle, amino acid metabolism, purine metabolism and fatty acid metabolism in the ESRD group. By contrast, the sarcopenia group with preserved renal function exhibited higher plasma levels of free fatty acids than control group. Pathway analysis of the metabolome data indicated enriched metabolite sets related to phospholipid biosynthesis in the sarcopenia group. In our proteomic analysis, plasma AHNAK protein was enriched in the sarcopenia group but depleted in the ESRD group, indicating potential differences in the mechanisms of muscle loss. A multi‐omic integration analysis revealed associations between relevant metabolites, including catecholamines, and a group of annotated proteins in extracellular exosomes.

Based on the metabolomic data, the ESRD group exhibited higher levels of epinephrine, norepinephrine and dopamine in the systemic circulation, which may lead to increased β‐adrenergic activation. Therefore, cardiovascular complications, including increased heart rate and systemic vascular resistance linked to β‐adrenergic activation, are common in patients with ESRD [14]. Moreover, β‐adrenergic activation can also impair renal function by modulating renin release, sodium retention and alterations in renal blood flow, leading to the development of hypertension‐mediated heart diseases [15, 16, 17]. The impact of catecholamines on skeletal muscle, which primarily expresses β2‐adrenergic receptors, has been explored [18]. Previous studies have indicated that the acute effects of catecholamines/β‐adrenergic agonists produce positive inotropic and lusitropic responses, leading to shortened relaxation periods. However, more recent studies have revealed that epinephrine‐induced lipolysis promotes futile and ATP‐consuming fatty acid–triglyceride cycling in burn patients, exacerbating muscle wasting [19, 20]. Interestingly, in this plasma metabolomic study, we identified a close association between β‐adrenergic activation and muscle wasting in ESRD group. Furthermore, we demonstrated that the primary difference in the mechanisms of primary sarcopenia and ESRD‐related muscle wasting could be β‐adrenergic activation. Thus, studying the use of β‐blockers in ESRD to mitigate the effects of increased sympathetic activity has attracted interest [21]. Although the decision to use β‐blockers in ESRD should be individualized, considering the patient's specific clinical condition, comorbidities and overall risk–benefit profile, further studies are required to establish the effect of β‐blockers on muscle mass and function and to identify subgroups who may benefit β‐blockers in patients with ESRD.

Many bile acid molecules are ranked among the top VIPs in the discriminant analysis of plasma metabolites in healthy controls and the sarcopenia group. MSEA data showed significant enrichment in phospholipid biosynthesis, including phosphatidylcholine and phosphatidylethanolamine, which corresponds well with the intergroup differences in bile acids, considering that phospholipids are critical components of bile. In addition to their classical functions, bile acids are known to function as metabolic regulators [22] and are associated with skeletal muscle function [23, 24]. The exercise‐inducible bile acid receptor, Takeda G protein‐coupled receptor 5, has been shown to improve skeletal muscle function in mice by promoting muscle cell differentiation and muscle hypertrophy [25]. Interestingly, glycolithocholic acid (GLCA) is the only bile acid that is more abundant in the circulation of patients with sarcopenia with the highest VIP score. A higher ratio of GLCA to chenodeoxycholic acid (the primary bile acid of GLCA) in plasma and stool has been reported to be independently associated with sarcopenia in cirrhosis [26]. Whether GLCA can serve as a common biomarker of chronic disease‐related muscle wasting needs further study. Additionally, ursodeoxycholic acid induces sarcopenia in mice and skeletal muscle impairment in cell culture models, leading to decreased protein synthesis and autophagic flux [27]. These findings suggest that bile acids can affect muscle function through various mechanisms, including the regulation of muscle cell differentiation, mitochondrial function and protein synthesis.

In the proteomic data, AHNAK showed a distinct pattern in the plasma among the groups; it was higher in the sarcopenia group and lower in the ESRD group than in the control group. AHNAK encodes for desmoyokin, whose carboxyl‐terminal domain was initially known to induce actin bundling and stabilizes muscle contraction [28]. In humans, AHNAK expression in skeletal muscle decreases with exercise training and increases with aging, and high AHNAK expression is associated with a low maximal oxygen uptake and poor muscle fitness [29]. In mice, the level of AHNAK protein in skeletal muscle is significantly upregulated with age, and the age‐related decline in muscle performance can be attenuated by reduced AHNAK expression [30]. AHNAK can also preferentially and strongly interact with phospholipids [31], which may be related to the phospholipid metabolism enriched in the metabolome analysis of the sarcopenia group. These reports align with the signature of AHNAK from primary sarcopenia in our study. A recent report provided evidence that AHNAK protein can be released from the muscle cells into the blood: In patients with limb girdle muscular dystrophy, AHNAK lost its sarcolemmal localization and was present outside the muscle fibre, a mislocalization due to the release of AHNAK‐containing vesicles from muscles [32]. In another report, the plasma proteome was compared between healthy individuals and patients with adolescent idiopathic scoliosis, and AHNAK was among the top major differential proteins related to this primary musculoskeletal disease [33], implying AHNAK as a blood biomarker related to muscle status. Interestingly, AHNAK is also closely related to sympathetic signalling, one of the predominant signatures in our metabolomic data from ESRD‐related muscle wasting. For example, in cardiomyocytes, AHNAK can act as a repressor towards Cav1.2 channel conductance, which can be disinhibited by phosphorylation of AHNAK upon sympathetic stimulation [34]. AHNAK deficiency in mice leads to increased responsiveness to β‐adrenergic signalling, resulting in browning and lipolysis in adipose tissues [35]. These examples suggest that AHNAK might be a negative regulator of sympathetic signalling in various cell types, which could be a potential therapeutic target for muscle loss in patients with ESRD. Whether decreased AHNAK in the plasma of patients with ESRD are mechanistically related to increased muscle wasting and decreased function requires further clarification.

In the ESRD group of current study, triggering receptors expressed on myeloid cells (TREML1), prostaglandin D2 synthase (PTGDS) and lipocalin‐1 were upregulated, whereas inter‐α‐trypsin inhibitor heavy chain 4 (ITIH4), oncostatin M receptor (OSMR), V‐set and immunoglobulin domain containing 4 (VSIG4), vitelline membrane outer layer protein 1 homologue and aminolevulinate dehydratase were downregulated compared with those in the control group. Some of the proteins here may be related to the haemodialysis or kidney, including TREML1 (exclusively expressed in the α‐granules of megakaryocytes and platelets and involved in platelet aggregation) [S1], PTGDS (contributing to progression of renal failure and dialysis dementia) [S2], ITIH4 (with the potency to suppress acute kidney injury) [S3] and VSIG4 (expressed not in skeletal muscle but in macrophage, which may act to alleviate renal tubulointerstitial injury) [S4]. Low OSMR level also may not be solely due to sarcopenia but related to ESRD or chronic disease status, considering its muscle‐specific deletion can even preserve muscle mass [S5]. Meanwhile, superoxide dismutase 2 (SOD2), PIK3IP1, AHCY, DBNL, TXN, CSF1 and HK1 were decreased in the sarcopenia group. SOD2 is highly expressed in the skeletal muscle, and its deletion affects specific aspects of muscle lipid metabolism, including the abundance of phospholipids and phosphatidic acid [S6], which correlates well with our metabolomic data in sarcopenia group. Among the top enrichment proteomic sets, hemopexin attracts our attention the most, as muscle atrophy‐induced hemopexin accelerated the onset of cognitive impairment in an Alzheimer's disease mouse model [S7]. Thus, the sets of proteins changed in ESRD and sarcopenia differ, indicating potential differences in the mechanisms of muscle loss. CNTFR, kininogen 1 (KNG1) and tropomodulin 4 (TMOD4) were decreased, whereas β2 microglobulin (B2M) and bromodomain PHD finger transcription factor (BPTF) were increased in the plasma of both sarcopenia and ESRD groups compared with the control group. KNG1, TMOD4 and B2M have been suggested as common disease‐independent atrophy markers in humans [S8].

Although no drugs for muscle wasting in ESRD have been reported as effective or approved [2], several clinical trials have been conducted to maintain muscle mass and function. Nandrolone decanoate, a synthetic derivative of testosterone, increases appendicular lean mass in patients undergoing dialysis, although the functional improvement was not statistically significant [36]. Phase I/II clinical trials evaluating anti‐myostatin peptibody (a chimeric peptide‐Fc fusion protein), PINTA 745, in patients with ESRD have revealed that LBM after 12 weeks did not meet the primary endpoint, and the development was halted. Four weeks of subcutaneous injection at each haemodialysis session using Anakinra, an interleukin‐1 receptor antagonist, did not show a significant benefit in LBM [37]. Adding recombinant human growth hormone to intradialytic parenteral nutrition and exercise protocol demonstrated a modest beneficial effect on whole‐body net protein balance [38]. Moreover, in patients with ESRD, treatment with calcitriol (active vitamin D) or paricalcitol increased cross‐section area of thigh‐muscle, as well as muscle strength and physical balance [39, 40]. However, because clinical trials based on multi‐omic data have not yet been conducted, new clinical trials aimed at reducing catecholamine effects may also be necessary.

The strength of our pilot study was the identification of potential metabolites and proteins in patients with ESRD with muscle loss and those with sarcopenia. This study also revealed possible metabolic pathways using differential analyses of the plasma metabolome and proteome, along with their integrative analysis. Moreover, this multi‐omic approach suggested a catecholamine activation of β‐adrenergic receptors as a potential candidate mechanism of haemodialysis‐mediated muscle wasting compared with sarcopenia with preserved renal function. However, this study has several limitations. First, the causality between the plasma levels of metabolites and proteins and sarcopenia could not be established, as this was a cross‐sectional study, warranting longitudinal studies to confirm these relationships. Second, we could not exclude the underlying diseases of the enrolled participants, such as diabetes, hypertension and dyslipidaemia or relevant potential confounders. Further studies with more rigorous control of data from patients with underlying conditions are needed to enhance the accuracy of the findings. Third, this pilot study may have had low statistical power due to the relatively small sample size. Further studies with larger, multicentre sample sizes are required to validate our findings and ensure their generalizability. Finally, the different age distributions used to match the degree of muscle loss and function among the three groups may limit the generalizability of our findings to other settings and populations. Muscle loss occurs more rapidly in patients undergoing dialysis than in healthy individuals, meaning that sarcopenia affects dialysis patients at an earlier age than in the general population. To account for this this, we aimed to match muscle mass and function across the groups, which resulted in age differences. In future studies, we need to consider more diverse age groups and additional demographic variables.

In conclusion, this comparative analysis of participants with sarcopenia and patients with ESRD using a multi‐omic approach provides new insights into the mechanisms underlying ESRD‐related muscle wasting that are distinct from those of primary sarcopenia. The findings may provide a basis for interventions in patients with ESRD with muscle loss and dysfunction. Moreover, this study contributes to the growing body of knowledge regarding muscle disorders, which may lead to the development of targeted interventions, personalized treatments and preventive strategies for managing muscle wasting in these patient populations. Further studies with larger sample sizes are required to validate our findings and elucidate the causal relationship between each metabolite and sarcopenia progression.

## Ethical Statement

This study was approved by the Institutional Review Board of Chungnam National University Hospital (CNUH‐2020‐10‐019‐005), and written informed consent was obtained from all the participants. The authors of this manuscript certify that they comply with the ethical guidelines for authorship and publishing in the Journal of Cachexia, Sarcopenia and Muscle.

## Conflicts of Interest

The authors declare no conflicts of interest.

## Supporting information


**Figure S1** Study enrolment flow chart. BIA, bioelectrical impedance analysis; ESRD, end‐stage renal disease.
**Figure S2.** Assessment of chemical‐protein interactions from metabolome and proteome data by MBROLE3. FDR, false discovery rate; HMDB, Human Metabolome Database; MeSH, Medical Subject Headings.

**Data S1** Supplementary Information.

**Table S1** Metabolomic profile of plasma from controls, participants with primary sarcopenia and patients with end‐stage renal disease–related muscle wasting.

**Table S2** Proteomic profile of plasma from controls, participants with primary sarcopenia and patients with end‐stage renal disease–related muscle wasting.
