# Supplementary material for: Comparative Analysis of Primary Sarcopenia and End‐Stage Renal Disease–Related Muscle Wasting Using Multi‐Omics Approaches
Source: J Cachexia Sarcopenia Muscle. 2025 Apr 10;16(2):e13749. doi: 10.1002/jcsm.13749 (PMC11982700; doi:10.1002/jcsm.13749)
Supplement: Supplementary file 1 — Figure S1 Study enrolment flow chart. BIA, bioelectrical impedance analysis; ESRD, end‐stage renal disease. Figure S2. Assessment of chemical‐protein interactions from metabolome and proteome data by MBROLE3. FDR, false discovery rate; HMDB, Human Metabolome Database; MeSH, Medical Subject Headings. [file JCSM-16-e13749-s001.docx]

***Journal of Cachexia Sarcopenia and Muscle***

***Original article***

- **Comparative analysis of primary sarcopenia and end-stage renal disease-related muscle wasting using multi-omics approaches**
- Daiki Setoyama^1,†^, Dohyun Han^2,†^, Jingwen Tian^3,4^, Ho Yeop Lee^3,4^, Hyun Suk Shin^2^, Ha Thi Nga^3,4^, Thi Linh Nguyen^3,4^, Ji Sun Moon^3^, Hyo Ju Jang^3,4^, Evonne Kim^5^, Seong-Kyu Choe^6,7^, Sang Hyeon Ju^8^, Dae Eun Choi^8^, Obin Kwon^9,10,^*, Hyon-Seung Yi^3,4,7,8,^*
- ^1^Department of Clinical Chemistry and Laboratory Medicine, Kyushu University Hospital, Fukuoka, Japan
- ^2^Proteomics Core Facility, Seoul National University Hospital Biomedical Research Institute, Seoul, South Korea
- ^3^Laboratory of Endocrinology and Immune System, Chungnam National University School of Medicine, Daejeon, South Korea
- ^4^Department of Medical Science, Chungnam National University School of Medicine, Daejeon, South Korea
- ^5^Department of Biomedical Sciences, BK21 FOUR Biomedical Science Program, Seoul National University College of Medicine, Seoul, South Korea
- ^6^Department of Medicine, Graduate School, Wonkwang University, Iksan, South Korea
- ^7^Sarcopenia Total Solution Center, Wonkwang University, Iksan, South Korea
- ^8^Department of Internal Medicine, Chungnam National University School of Medicine, Daejeon, South Korea
- ^9^Department of Biochemistry and Molecular Biology, Seoul National University College of Medicine, Seoul, South Korea
- ^10^Genomic Medicine Institute, Medical Research Center, Seoul National University, Seoul, South Korea

^†^These authors contributed equally to this article.

***Correspondence to:**HSY (jmpbooks@cnu.ac.kr); Tel: +82-42-280-6994; Fax: +82-42-280-7995
OK (obinkwon@snu.ac.kr); Tel: +82-2-740-8240; Fax: +82-2-3668-7897

**Supplementary methods**

*Extraction and LC-MS****-based measurements*** *of plasma water-soluble metabolites, acylcarnitines, and glycerophospholipids*

To comprehensively extract water-soluble metabolites, acylcarnitines, and phospholipids from human plasma, 10 μL of the sample was combined with 40 μL of water and 250 μL of ice-cold methanol. The mixture was vigorously vortexed, followed by centrifugation at 21,500 g for 5 min at 4 °C. The resulting supernatant was collected and combined with 150 μL of water, constituting the direct sample for acylcarnitine analysis. For water-soluble metabolites, a 3-fold dilution in 0.1% formic acid water was used. In addition, a 50-fold dilution in 0.1% formic acid water was used for phospholipid analysis. Acylcarnitine analysis was performed using a triple quadrupole mass spectrometer (LCMS-8060, Shimadzu), and HILIC chromatography with a Luna 3u HILIC 200A column (150×2.0 mm, 3 µm particle size, Phenomenex) was employed to monitor **37** acylcarnitine **species**. The mobile phase consisted of solvent A (10 mM ammonium formate) and solvent B (acetonitrile:10 mM ammonium formate, 9:1), and the column oven temperature was 40 ℃. The gradient elution program was as follows: a flow rate of 0.3 mL/min: 0–2.5 min, 100% B; 2.5–4 min, 100%–50% B; 4–7.5 min, 50%–5% B; 7.5–10 min, 5% B; and 10.1–12.5 min, 100% B. The parameters for the heated ESI in the positive ion mode under precursor ion scan were as follows: drying gas flow rate, 10 L/min; nebulizer gas flow rate, 3 L/min; heating gas flow rate, 10 L/min; interface temperature, 300 °C; DL temperature, 250 °C; heat block temperature, 400 °C; CID gas, 270 kPa. Acylcarnitine profiles were detected under precursor ion scan for m/z 85.5 by changing the CE according to the length of the fatty acids: -20 for short-chain acylcarnitines (C0–C8); -35 for middle-chain acylcarnitines (C9–C12); -45 for long-chain acylcarnitineC12–C18. To measure **107** water-soluble metabolites, the sample was separated on a Discovery HS-F5-3 column (150×2.1 mm, 3 μm particle size, Sigma-Aldrich) with mobile phases consisting of solvent A (0.1% formic acid) and solvent B (0.1% formic acid in acetonitrile). The column oven temperature was 40℃. The gradient elution program was as follows: a flow rate of 0.25 mL/min: 0–2 min, 0% B; 2–5 min, 0%–25% B; 5–11 min, 25%–35% B; 11–15 min, 35%–95% B; 15–25 min, 95% B; 25.1–30 min, 0% B. The parameters for the heated electrospray ionization source (ESI) in negative/positive ion mode under multiple reaction monitoring (MRM) were as follows: drying gas flow rate, 10 L/min; nebulizer gas flow rate, 3 L/min; heating gas flow rate, 10 L/min; interface temperature, 300 ̊C; DL temperature, 250 ̊C; and heat block temperature, 400 ̊C; CID gas, 270 kPa. For glycerophospholipids, the prepared sample was separated on a Kinetex C8 column (150×2.1 mm, 2.6 μm particle size, Phenomenex) with mobile phases consisting of solvent A (20mM ammonium formate) and solvent B [acetonitrile/isopropanol (1:1, v/v)]. The column oven temperature was 45℃. The gradient elution program was as follows: flow rate of 0.3 mL/min: 0–1 min, 20% B; 1–2 min, 40% B; 2–25 min, 92.5% B; 25.1–35 min, 100% B; 35.1–38 min, 20% B. **Profiling of 96 phospholipids was measured** using the ESI-positive ion mode under MRM.

*Extraction and LC-MS****-based measurements*** *of plasma-free fatty acids and bile acids*

To extract free fatty acids and bile acids from human plasma, 30 μL of the sample was combined with 120 μL of ice-cold methanol. The mixture was vigorously vortexed, followed by centrifugation at 21,500 g for 5 min at 4 °C. The resulting supernatant was collected, constituting a direct sample for free fatty acid analysis. To prepare for the bile acid measurement, 40 uL of the supernatant was evaporated and dissolved in 40 μL of 20% methanol. For free fatty acids, the sample was separated on an ACQUITY BEH Amide column (150 mm×2.1 mm, 1.7 μm, waters) with mobile phases consisting of solvent A (10 mM ammonium formate in 90% acetonitril) and solvent B (0.1% formic acid in acetonitrile). The column oven temperature was 40ºC. The gradient elution program was as follows: a flow rate of 0.4 mL/min, 0–5 min, 0% B; 5.1–7.5 min, 100% B; 7.6–11 min, 0% B. **Profiles of 10** free fatty acid profiles **was measured** using the ESI negative ion mode under selected ion monitoring (SIM). For bile acids, the sample was separated on an ACQUITY BEH Amide column (150 mm×2.1 mm, 1.7 μm, waters) with mobile phases consisting of solvent A (0.1% formic acid) and solvent B (acetonitrile). The column oven temperature was 50℃. The gradient elution program was as follows: a flow rate of 0.3 mL/min: 0–2 min, 20% B; 2–10 min, 80% B; 10–12 min, 80% B; 12.1–15 min, 20% B. **Profiles of 19** bile acids **was measured** using the ESI negative ion mode under MRM.

*Sample preparation for plasma proteomics*

To remove high-abundance proteins, 30 µL of plasma samples were diluted 1:4 with multiple affinity removal system (MARS) buffer A (Agilent Technologies, Santa Clara, CA, USA) and filtered with 0.22 μm Spin-X filters (Corning Costar, NY, USA). Individual plasma samples were depleted of 14 high-abundance human blood proteins [albumin, Immunoglobulin (Ig) G, IgA, transferrin, haptoglobin, fibrinogen, α2-macroglobulin, α1-acid glycoprotein, IgM, apolipoprotein AI, apolipoprotein AII, complement C3, and transthyretin] using a MARS column (Hu-14HC, 4.6 × 100 mm, Agilent Technologies, Santa Clara, CA, USA) on an Agilent 1260 bionert HPLC system. Depleted plasma samples were concentrated by centrifugal filtration using a 3 kDa Amicon filter (Millipore, Burlington, MA, USA). Protein concentration was measured using the Bicinchoninic acid (BCA) assay.

Prior to protein digestion, 100 µg of depleted samples were precipitated by adding a 5-fold volume of ice-cold acetone. The precipitated samples were reconstituted in 50 µL of SDT buffer (2% sodium dodecyl sulfate, 0.1 M dithiothreitol in 0.1 M Tris HCl pH 8.0). To denature proteins, samples were heated for 20 minutes at 95 °C and subsequently digested using a filter-aided sample preparation (FASP) method, as previously described with some modifications.^1^ Briefly, protein samples were loaded onto a 30 K amicon filter (Millipore, Billerica, MA, USA), and the buffer was exchanged with UA solution (8 M urea in 0.1 M Tris-HCl pH 8.5) via centrifugation. After three buffer exchanges with UA solution, the reduced cysteines were alkylated with 0.05 M iodoacetamide in UA solution for 30 min in the dark at room temperature. Thereafter, the UA buffer was exchanged twice with 40 mM ammonium bicarbonate (ABC). Protein samples were digested with trypsin/LysC (enzyme to substrate ratio of 1:100) at 37 °C for 16 h. The resulting peptides were collected in new Eppendorf tubes via centrifugation, and an additional elution step was performed using 40 mM ABC and 0.5 M NaCl. All resulting peptides were acidified with 10% trifluoroacetic acid and desalted using in-house C18-StageTips, as described previously.^1^ The desalted peptides were completely dried in a vacuum dryer and stored at − 80 °C for further analysis.

*LC-MS/MS-based proteome measurements*

Liquid Chromatography with tandem mass spectrometry (LC-MS/MS) analysis was performed using quadrupole Orbitrap mass spectrometers, Q-exactive plus (Thermo Fisher Scientific, Waltham, MA, USA), coupled to an Ultimate 3000 RSLC system (Dionex, Sunnyvale, CA, USA) with a nano-electrospray source as previously described, with some modifications.^2^ Peptide samples were separated on a two-column setup with a trap column (300 μm I.D. × 5 mm, C18 3 μm, 100 Å) and an analytical column (75 μm I.D. × 50 cm, C18 1.9 μm, 100 Å). Prior to sample injection, the dried peptide samples were re-dissolved in solvent A (2% acetonitrile and 0.1% formic acid). After the samples were loaded onto the nano LC, a 120-min gradient from 8 to 30% solvent B (100% acetonitrile and 0.1% formic acid) was applied to all samples. The spray voltage was 2.0 kV in positive ion mode, and the temperature of the heated capillary was set to 320 °C. The hyperreaction monitoring (HRM) data-independent acquisition (DIA) method consisted of a survey scan at 70,000 resolution from 400 to 1,220 m/z (AGC target of 3 × 106 and 60 ms injection time). Further, 19 dynamic DIA windows were acquired at a resolution of 35,000 with an automatic gain control target of 3e6 and auto injection time.^3^ The stepped collision energy was 10% at 27% (25, 27, and 29%).

*Proteomics data processing and analysis*

To generate spectral libraries, 24 data-dependent acquisition (DDA) measurements were performed on the depleted pooling plasma samples. The DDA data were imported to Spectronaut to generate the DDA library by Pulsar with default settings against the UniProt Human Database (July 2021, 101,014 entries) and the indexed retention time standard peptide sequence. DIA data from Individual samples were analyzed using Spectronaut version 17 (Biognosys, Schlieren-Zurich, Switzerland). First, the DIA raw files were converted into HTRMS format using the GTRMS converter tool provided by Spectronaut. The false discovery rate was estimated using the mProphet approach and set to 1% at the peptide precursor and protein levels.^4^ The proteins were inferred by the software, and the quantification information was acquired at the protein level using a q-value < 0.01 criterion, which was used for the subsequent analyses.

**References for Supplementary methods**

1. Kim SI, Hwangbo S, Dan K, Kim HS, Chung HH, Kim JW, et al. Proteomic Discovery of Plasma Protein Biomarkers and Development of Models Predicting Prognosis of High-Grade Serous Ovarian Carcinoma. *Mol Cell Proteomics* 2023;**22**:100502.

2. Jeong HY, An HJ, Sung MJ, Ha MH, Lee YH, Yang DH, et al. Proteomic profiling of protein expression changes after 3 months-exercise in ESRD patients on hemodialysis. *BMC Nephrol* 2023;**24**:102.

3. Bruderer R, Bernhardt OM, Gandhi T, Miladinovic SM, Cheng LY, Messner S, et al. Extending the limits of quantitative proteome profiling with data-independent acquisition and application to acetaminophen-treated three-dimensional liver microtissues. *Mol Cell Proteomics* 2015;**14**:1400-1410.

4. Reiter L, Rinner O, Picotti P, Huttenhain R, Beck M, Brusniak MY, et al. mProphet: automated data processing and statistical validation for large-scale SRM experiments. *Nat Methods* 2011;**8**:430-435.

**Supplementary Figures**

**
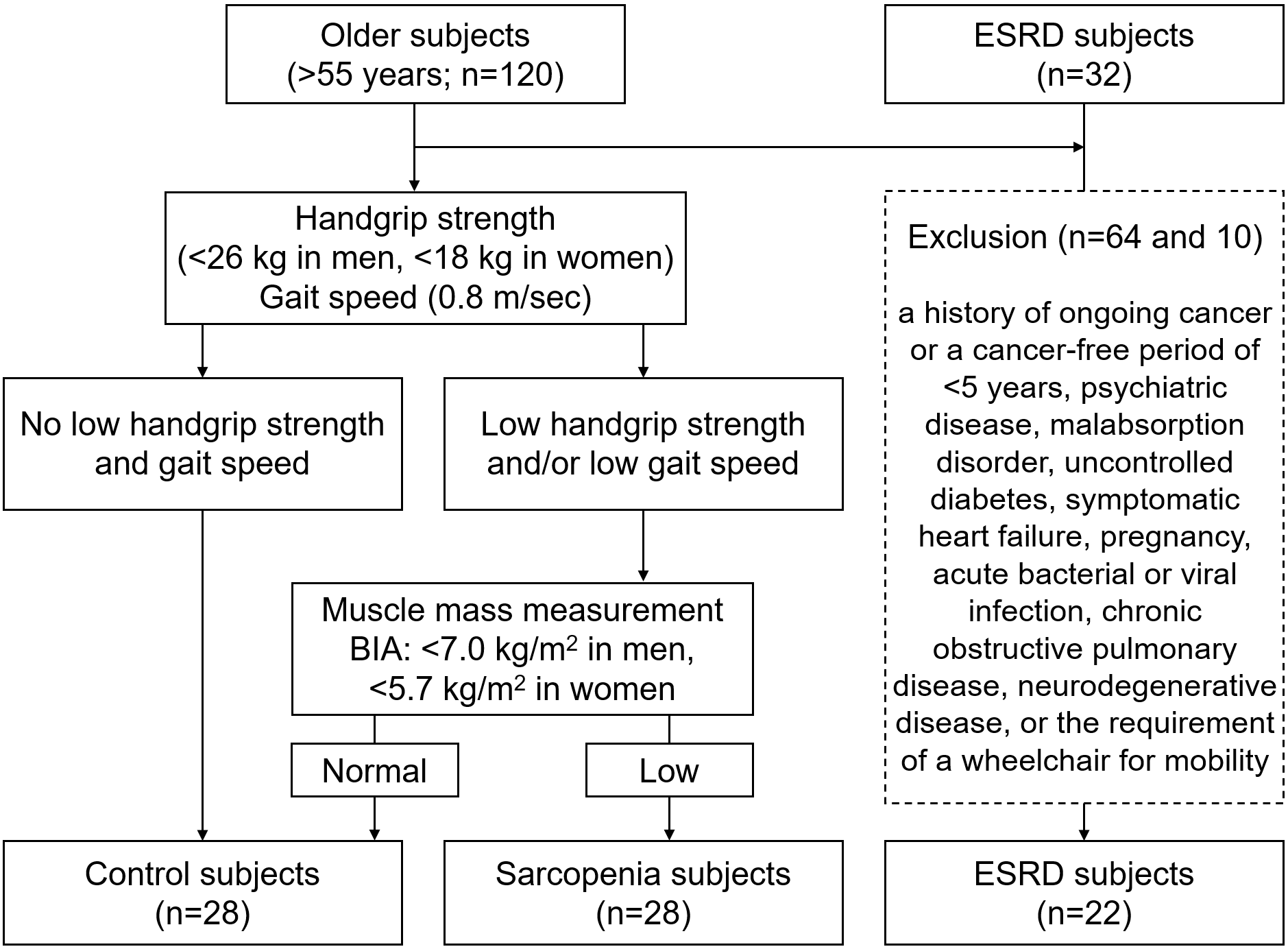
**

**Figure S1. Study enrollment flow chart. BIA, bioelectrical impedance analysis; ESRD, end-stage renal disease.**

**
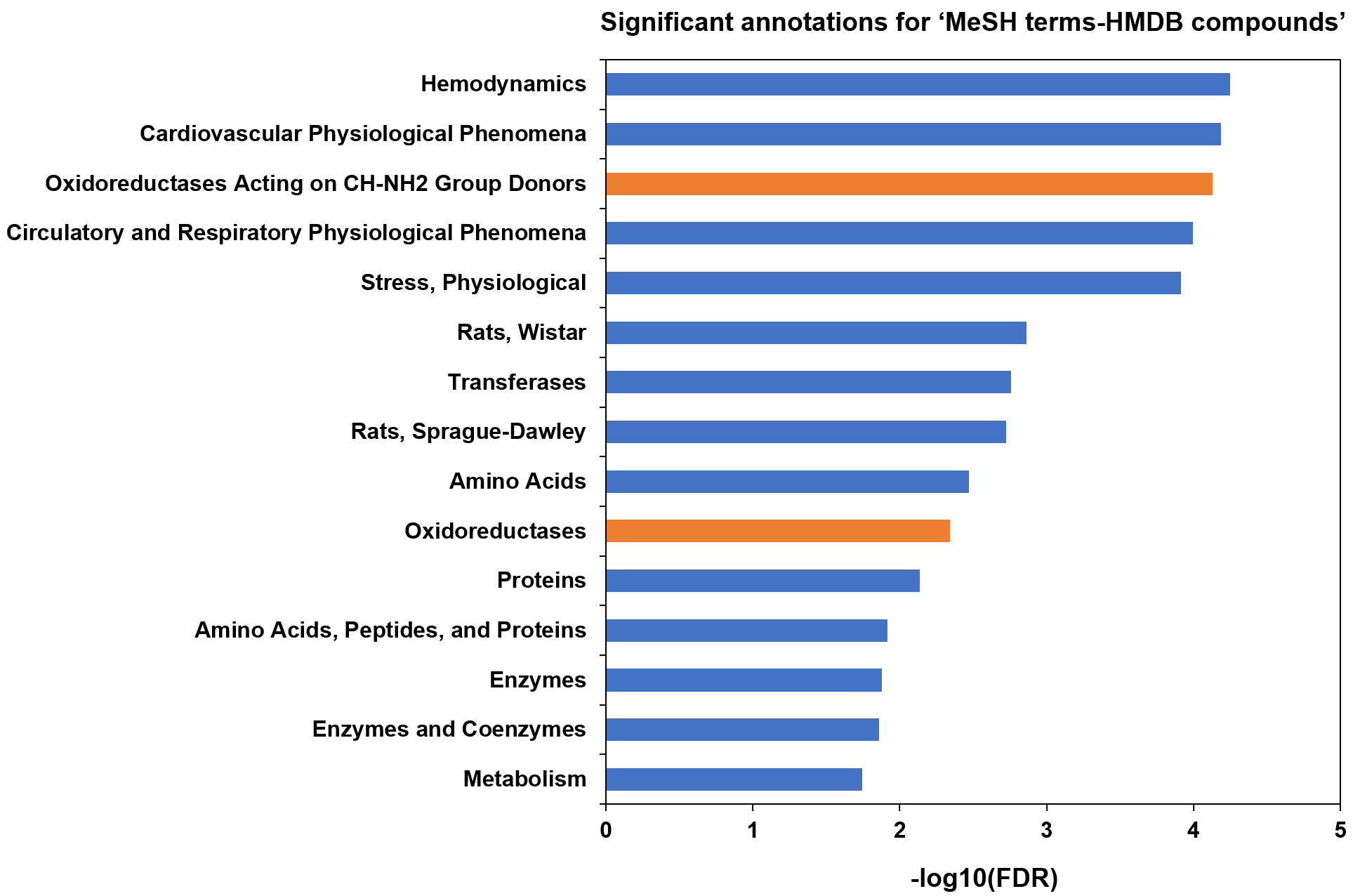
**

**Figure S2. Assessment of chemical-protein interactions from metabolome and proteome data by MBROLE3. FDR, false discovery rate; HMDB, Human Metabolome Database; MeSH, Medical Subject Headings.**

**Supplementary Tables**

**Table S1. Metabolomic profile of plasma from controls, participants with primary sarcopenia, and patients with end-stage renal disease-related muscle wasting**

**Table S2. Proteomic profile of plasma from controls, participants with primary sarcopenia, and patients with end-stage renal disease-related muscle wasting**
