# Supplementary material for: Comparative Analysis of Primary Sarcopenia and End‐Stage Renal Disease–Related Muscle Wasting Using Multi‐Omics Approaches
Source: J Cachexia Sarcopenia Muscle. 2025 Apr 10;16(2):e13749. doi: 10.1002/jcsm.13749 (PMC11982700; doi:10.1002/jcsm.13749)
Supplement: Supplementary file 2 — Data S1 Supplementary Information. [file JCSM-16-e13749-s002.docx]

- **Comparative analysis of primary sarcopenia and end-stage renal disease-related muscle wasting using multi-omics approaches**
- Daiki Setoyama^1,†^, Dohyun Han^2,†^, Jingwen Tian^3,4^, Ho Yeop Lee^3,4^, Hyun Suk Shin^2^, Ha Thi Nga^3,4^, Thi Linh Nguyen^3,4^, Ji Sun Moon^3^, Hyo Ju Jang^3,4^, Evonne Kim^5^, Seong-Kyu Choe^6,7^, Sang Hyeon Ju^8^, Dae Eun Choi^8^, Obin Kwon^9,10,^*, Hyon-Seung Yi^3,4,7,8,^*

***Correspondence to:**HSY (jmpbooks@cnu.ac.kr); Tel: +82-42-280-6994; Fax: +82-42-280-7995
OK (obinkwon@snu.ac.kr); Tel: +82-2-740-8240; Fax: +82-2-3668-7897

**Supplementary references**

S1. Colonna M. The biology of TREM receptors. *Nat Rev Immunol* 2023;**23**:580-594.

S2. Maesaka JK, Palaia T, Fishbane S, Ragolia L. Contribution of prostaglandin D2 synthase to progression of renal failure and dialysis dementia. *Semin Nephrol* 2002;**22**:407-414.

S3. Huo Y, Lai Y, Feng Q, Wang Q, Li J. Serum ITIH4 in coronary heart disease: a potential anti-inflammatory biomarker related to stenosis degree and risk of major adverse cardiovascular events. *Biomark Med* 2022;**16**:1279-1288.

S4. Li Y, Wang YQ, Wang DH, Hou WP, Zhang Y, Li M, et al. Costimulatory molecule VSIG4 exclusively expressed on macrophages alleviates renal tubulointerstitial injury in VSIG4 KO mice. *J Nephrol* 2014;**27**:29-36.

S5. Domaniku-Waraich A, Agca S, Toledo B, Sucuoglu M, Ozen SD, Bilgic SN, et al. Oncostatin M signaling drives cancer-associated skeletal muscle wasting. *Cell Rep Med* 2024;**5**:101498.

S6. Zhuang A, Yang C, Liu Y, Tan Y, Bond ST, Walker S, et al. SOD2 in skeletal muscle: New insights from an inducible deletion model. *Redox Biol* 2021;**47**:102135.

S7. Nagase T, Tohda C. Skeletal muscle atrophy-induced hemopexin accelerates onset of cognitive impairment in Alzheimer's disease. *J Cachexia Sarcopenia Muscle* 2021;**12**:2199-2210.

S8. Aniort J, Stella A, Philipponnet C, Poyet A, Polge C, Claustre A, et al. Muscle wasting in patients with end-stage renal disease or early-stage lung cancer: common mechanisms at work. *J Cachexia Sarcopenia Muscle* 2019;**10**:323-337.
